# Increasing the Power to Detect Causal Associations by Combining Genotypic and Expression Data in Segregating Populations

**DOI:** 10.1371/journal.pcbi.0030069

**Published:** 2007-04-13

**Authors:** Jun Zhu, Matthew C Wiener, Chunsheng Zhang, Arthur Fridman, Eric Minch, Pek Y Lum, Jeffrey R Sachs, Eric E Schadt

**Affiliations:** 1 Rosetta Inpharmatics, Seattle, Washington, United States of America; 2 Department of Applied Computer Science and Mathematics, Merck Research Laboratories, Rahway, New Jersey, United States of America; Washington University, United States of America

## Abstract

To dissect common human diseases such as obesity and diabetes, a systematic approach is needed to study how genes interact with one another, and with genetic and environmental factors, to determine clinical end points or disease phenotypes. Bayesian networks provide a convenient framework for extracting relationships from noisy data and are frequently applied to large-scale data to derive causal relationships among variables of interest. Given the complexity of molecular networks underlying common human disease traits, and the fact that biological networks can change depending on environmental conditions and genetic factors, large datasets, generally involving multiple perturbations (experiments), are required to reconstruct and reliably extract information from these networks. With limited resources, the balance of coverage of multiple perturbations and multiple subjects in a single perturbation needs to be considered in the experimental design. Increasing the number of experiments, or the number of subjects in an experiment, is an expensive and time-consuming way to improve network reconstruction. Integrating multiple types of data from existing subjects might be more efficient. For example, it has recently been demonstrated that combining genotypic and gene expression data in a segregating population leads to improved network reconstruction, which in turn may lead to better predictions of the effects of experimental perturbations on any given gene. Here we simulate data based on networks reconstructed from biological data collected in a segregating mouse population and quantify the improvement in network reconstruction achieved using genotypic and gene expression data, compared with reconstruction using gene expression data alone. We demonstrate that networks reconstructed using the combined genotypic and gene expression data achieve a level of reconstruction accuracy that exceeds networks reconstructed from expression data alone, and that fewer subjects may be required to achieve this superior reconstruction accuracy. We conclude that this integrative genomics approach to reconstructing networks not only leads to more predictive network models, but also may save time and money by decreasing the amount of data that must be generated under any given condition of interest to construct predictive network models.

## Author Summary

Complex phenotypes such as common human diseases are caused by variations in DNA in many genes that interact in complex ways with a number of environmental factors. These multifactorial gene and environmental perturbations induce changes in molecular networks that in turn lead to phenotypic changes in the organism under study. The comprehensive monitoring of transcript abundances using gene expression microarrays in different tissues over a large number of individuals in a population can be used to reconstruct molecular networks that underlie higher-order phenotypes such as disease. The cost to generate these large-scale gene activity measurements over large numbers of individuals can be extreme. However, by integrating DNA variation and gene activity data monitored in each individual in a given population of interest, we demonstrate that the power to elucidate molecular networks that drive complex phenotypes can be significantly enhanced, without increasing the sample size. Using a biologically realistic simulation framework, we demonstrate that molecular networks reconstructed using the combined DNA variation and gene activity data are more accurate than molecular networks reconstructed from gene activity data alone, implying that adding DNA variation data might allow us to use fewer subjects to produce molecular networks that better explain complex phenotypes such as disease.

## Introduction

Normal physiology, disease processes, and response to drug treatment all involve complex interactions among genes and between genes and environmental factors. New high-throughput functional genomics technologies such as gene expression microarrays provide an enormous amount of data on how genes respond to genetic and environmental perturbations. Network and pathway methods, in which nodes represent genes and edges (links) between two nodes indicate a relationship between the corresponding genes, provide a useful framework for extracting and organizing information from such data. One of the primary aims in reconstructing reliable gene networks is to predict which genes respond directly to a stimulus (primary gene changes), as opposed to those genes that respond to changes in the primary genes (secondary gene changes).

Some network reconstruction methods are based on pairwise relationships among genes, while others, such as Bayesian network reconstruction methods, explicitly examine interactions involving more than two genes, attempting to separate direct from indirect influences. For example, an edge between two genes may indicate that the corresponding expression traits are correlated in a population of interest, or it may indicate that changes in the activity of one gene led to changes in the activity of the other gene [1]. Ideally, a network will allow us to predict the system's response (or the probability of various responses) to any given perturbation.

Here we represent biological networks of genes as Bayesian networks [2], which have successfully represented some biological systems [3,4]. The edges in Bayesian networks have direction, and the topology of a Bayesian network defines certain relationships among the nodes. That is, given the states of the parent nodes—the nodes with edges that point to a node of interest—you can predict (probabilistically) the state of a node of interest. Cycles—paths that return to a starting node—are not allowed, meaning that certain types of feedback cannot be represented by Bayesian networks. Ideally, we would like to find the network that best explains the observed data, in the sense of maximizing a probability function on the network given the data (see Methods), but this presents several problems. First, the number of possible networks grows rapidly with the number of genes under consideration. This makes it impossible to examine all possible networks, so heuristic searches are used. Second, even if we could examine all possible networks, we face an underdetermined problem: the number of samples available in most microarray experiments is much smaller (often orders of magnitude smaller) than the number of genes, so many networks explain the observed data equally well. In particular, because Bayesian networks represent multivariate probability distributions (see Methods), the direction of many of the edges in such networks can be changed without affecting how well the model fits the data (Markov equivalence). Thus, both the data and the reconstruction method limit our ability to make inferences about causal relations among genes.

These limitations raise the question of whether and how network reconstruction can be improved by including other types of data. In segregating populations arising naturally or from experimental crosses, genetic information (e.g., genotypic data) can provide important information about which genes interact and can identify the relationships among interacting genes [5]. Different alleles for a given gene are often associated with systematic differences in transcript abundances for the gene, as has been shown in several species [5–9]. In the context of segregating populations, significant differences between allele-specific transcript levels can be detected as expression quantitative trait loci (eQTL) [9,10]. Gene expression traits driven by common eQTL provide the structural information needed to identify which genes are likely to influence other genes, and this information can be used to bias the search for relationships among gene expression traits and between gene expression and other complex traits [1,9]. Importantly, the genetic data provide information as to which of a pair of interacting genes is causal (upstream) and which is reactive (downstream). Therefore, links in the reconstructed networks that are based on genotypic data have much stronger indications of causality than links based only on correlation information.

We have previously demonstrated that a network reconstructed using both gene expression and genetic information allows better prediction of the effect of experimental perturbation of a particular gene [5] than a network reconstructed using gene expression alone. Here we more formally assess the utility of integrating genotypic data to reconstruct gene networks by simulating genetic and gene expression data from biologically realistic networks and by quantifying the improvement in network reconstruction achieved using the combined data, compared with reconstruction using gene expression data alone. By reconstructing networks based on simulated datasets in which the number of samples was allowed to vary, we are able to estimate the incremental benefit of collecting additional data, in addition to the benefit of incorporating genotypic data. We conclude that our integrative genomics approach to reconstructing networks not only leads to more predictive network models, but may provide savings of time and money by decreasing the amount of data that must be generated under any given condition of interest to achieve a desired level of accuracy.

## Results

**Figure 1:**
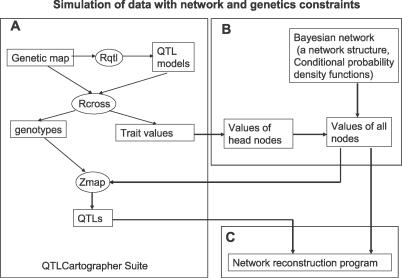
The Data Simulation Scheme with Genetic and Network Constraints (A) A segregating population (an F2 intercross in this case) is simulated using the QTL Cartographer software suite (Rqtl, Rcross, and Zmapqtl). The QTL model for a trait is defined using the Rqtl program, and the heritability of the QTL is defined using the Rcross program. (B) The traits simulated by Rcross are used as the head nodes in the simulated network. The remaining traits are simulated based on the values of the head nodes according to the DAG structure and the set of conditional probability density functions associated with this structure. (C) After traits for all nodes in the network are simulated, they are scanned for QTLs using the Zmapqtl program. The traits and the associated QTL are then input into the network reconstruction program.

Data were simulated following the scheme shown in Figure 1 (see Methods for details), using the Bayesian network structure derived from the BXD cross [5] as the true network, referred to here as the BXD network. The BXD network comprises 2,169 nodes (genes) and 1,676 directed connections, with 639 genes represented as singleton nodes (nodes with no connections to other nodes in the network). The general features of the BXD network, such as in-degree, out-degree, and connectivity distributions, are shown in Figure S1. The in-degree (out-degree) of a node is equal to the number of inward-directed (outward-directed) edges connected to the node, while the connectivity of a node (its degree) is equal to the number of edges connecting to the node. We also created a simple structure, referred to as the synthetic network, to allow comparison with previous results on network reconstruction accuracy obtained using networks with a small number of nodes. The synthetic network is an agglomeration of isolated three-node substructures (Figure S2). The synthetic structure has 2,160 nodes and 1,440 interactions, similar to the BXD network. More comprehensive examination of the effect of network structure on reconstruction accuracy and the use of genetic data is outside the scope of this study and will be explored in future work.

### Simulation of Genetic and Gene Expression Data

Figure 1 outlines the procedure used to simulate the genetic and gene expression data, given a particular network structure. First, we simulated an F2 intercross population of 1,000 individuals so that each individual in the population had a unique genetic background derived from two inbred mouse strains (Figure 1A). Second, we simulated the gene expression profile for each individual in the population, using the network structure and individual genotypes, providing the constraints necessary to achieve correlated expression and QTL structures in the simulated data (Figure 1B). See Methods for details.

We assumed a single eQTL for each head node in the network, and these eQTLs were evenly distributed over the 19 chromosomes. Given the genetic map, genotypes, and simulated expression values for the different genes, we used standard interval mapping procedures [11] to map eQTL for each of the simulated gene expression traits.

### Cis-/Trans-Acting eQTLs and Network Simulation

In experimental crosses, the source of systematic perturbations needed to reconstruct networks is identifiable: variations in DNA can lead to changes in expression, which are detected as eQTL. If DNA variation within the structural gene itself affects the expression of the gene, then we say that the DNA variation is cis-acting with respect to the gene and gives rise to a cis eQTL. Because genes with cis-acting eQTL directly reflect the source of systematic perturbations (DNA variations), they appear as head nodes in the Bayesian networks [5].

Gene expression values for the head nodes were constrained by the percent of total variation explained by genetic (eQTL) effects (i.e., narrow sense heritability). The genetic component of expression of all non-head nodes was then derived from the head nodes and network structure, resulting in the non-head nodes giving rise to trans eQTLs. Finally, starting with the simulated expression values at the head nodes and the conditional probabilities from the Bayesian network, expression values for the rest of the nodes were generated probabilistically, as described in Equations 1 and 2 (see Methods). These simulated data were used to construct a Bayesian network, as shown in Figure 1C, and the constructed network was then compared with the true network from which the data were simulated.

### Question I: How Faithfully Do We Reconstruct a Biologically Realistic Network?

To address the question of reconstruction accuracy, we simulated a dataset based on a known network, and compared the reconstructed network with the known network. As noted above, to make the simulations as biologically plausible as possible, we based network structure, the relations among different genes, and the degree of genetic heritability on the previously described BXD network, which was reconstructed from actual biological data collected in an F2 intercross population.

**Figure 2:**
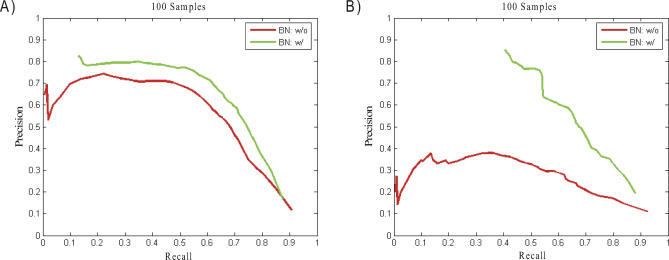
Reconstruction Accuracy Based on 100-Sample Datasets Generated Using Parameters Similar to BXD Data All accuracies are based on directed graphs unless indicated otherwise. (A) Accuracy of reconstructions with and without genetic information used as prior information. (B) Accuracy of reconstructions for the top-layer subnetwork, as defined in the text.

The average genetic heritability for the head nodes with cis-acting QTLs in the BXD data was 0.5. Correlation between gene expression levels for interacting genes tended to be high in this dataset (Figure S3A). The correlation distribution from this dataset was used to simulate a set of data comprising 100 samples. QTLs were then mapped for each node using a standard interval mapping method [11]. The distribution of QTL peaks is shown in Figure S3B. The QTL peaks for head nodes are evenly distributed along the chromosomes. The QTL peaks for all nodes are clustered into several hot spots, as was observed in the BXD data [9]. The ROC (receive operating curve)-like plots shown in Figure 2 demonstrate that a Bayesian network reconstructed with genetic information is more accurate than one constructed without genetic information. Each curve represents results from varying the consensus threshold; that is, the threshold for the number of individual MCMC networks in which an edge must be present to be included in the final reconstruction. The improvement in accuracy is relatively small for the full network (Figure 2A), but quite pronounced for the top layer of the network, where the top layer of the network is defined as the head nodes and their children (Figure 2B). For example, the network reconstructed with the genetics data achieved nearly 80% precision when recall was 50%, compared with 35% for the network reconstructed without genetic data.

If we ignore edge direction, we can compare the Bayesian network to a correlation-based association network reconstructed from the same data. The correlation-based network was reconstructed as previously described [12] (see Methods for details). As shown in Figure S7, the Bayesian networks with and without the genetic information used as prior information are more accurate than the correlation-based association network, whether we consider only direct connections (dashed lines) or an edge as correct if the corresponding genes are connected by either one or two edges in the true network (solid lines). In this more general sense, the accuracy of the Bayesian network with genetic information recovers 80% of the actual interactions, where 85% of the identified interactions are correct (at an edge inclusion threshold of 30%; green dot in Figure S7).

From these simulations, we noted that the improvements realized using genetic data were smaller than improvements we had previously seen in actual biological networks comprising only a handful of nodes and connections (e.g., simple three-node networks [1]). Therefore, a “synthetic network,” an agglomeration of isolated three-node substructures (Figure S2), was used to investigate this discrepancy. The synthetic structure comprised 2,160 nodes and 1,440 interactions, similar to the BXD network, but its connectivity structure was very different. For the synthetic structure, we assumed that all head nodes (1/3 of the nodes) gave rise to cis-acting eQTLs. For the simulated genetic data, heritability was again set to 0.5, which is the mean heritability for all genes with cis-acting eQTLs in the BXD cross. To simulate the data for the synthetic network, a population size of 100 was used, which again is similar to the size of the BXD cross (comprising 111 mice). The correlation coefficients between interacting genes were drawn from a normal distribution with mean 0.45 and standard deviation 0.1. The correlation coefficient cutoff for a random association in the network of this size is approximately 0.45, so that one-half of the interaction strengths is above the cutoff for random associations, while the other half is below the cutoff.

**Figure 3:**
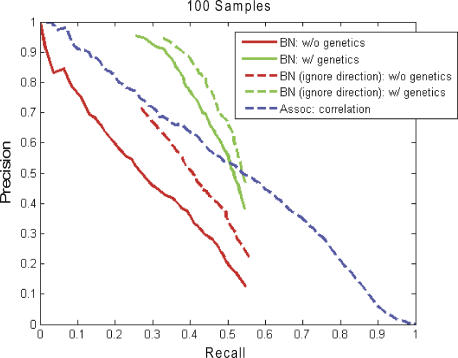
The Accuracy of Reconstruction of the Synthetic Network, Reconstructed with and without Genetic Information The genetics information not only helps to infer the direction of the relationships between nodes (solid lines), but also increases the power to detect relationships when direction is ignored, as with the association networks (dashed lines).

Figure 3 shows that the genetic data had a much larger effect on Bayesian network reconstruction accuracy for the synthetic network than for the BXD network. The improvement is substantial even if edge direction is ignored (dashed lines), which demonstrates that genetic information not only helps establish edge direction (causality), but also helps to pull out relations between genes even when causality cannot be unambiguously established. Interestingly, the network based purely on correlation information is more accurate than the Bayesian network constructed without genetic data, but less accurate than the Bayesian network constructed with the genetic data. The most reasonable explanation for this relates to the discretization process applied to the expression data. The continuous gene expression traits were discretized into one of three possible states (upregulated, downregulated, or not significantly upregulated or downregulated) guided by k-means clustering (see Methods for details), before they were input into the Bayesian network reconstruction program. The discretization process results in information loss in the input set of expression traits, and this loss in turn affects the reconstruction accuracy. On the other hand, no such information loss occurs in the input gene expression trait data used in the correlation-based network reconstruction. As can be seen from the ROC curves depicted in Figure S7, when the overall interaction strength in the underlying data was simulated to be high, the Bayesian network had an advantage over the simple correlation-based methods. However, when the correlation strength is low, the information loss due to discretization is larger than the gain realized from the Bayesian network method. The information loss realized in the discrete Bayesian network reconstruction notwithstanding, the advantages of discrete Bayesian networks over continuous Bayesian networks (where the continuous expression data are used in the reconstruction process) derives from their ability to represent nonlinear interactions and from increased processing efficiency, as described in Methods.

The increased effect of genetic data in the synthetic structure is likely due to a greater proportion of the head nodes giving rise to strong genetic effects (approximately 1/3 of the nodes instead of 1/20). Schadt et al. [1] demonstrated that it is possible to order traits by comparing independent, causal, and reactive models when the perturbation source (polymorphism at a locus) is known, where the model best supported by the data is chosen as the most likely relationship between the traits of interest. Genetic information at a node is most helpful in defining relationships with other genes directly connected to that node, and in general becomes less powerful as it is propagated through more nodes. Therefore, with more head nodes we expect greater improvement given the genetic information. The effect of genetic information may also depend on network structure and remains to be explored.

### Question II: Is the Power to Detect Both Weak and Strong Interactions Enhanced in the Presence of Strong (High-Heritability) Genetic Information? Do Genetics Still Improve Reconstruction Accuracy if Interactions Are Weaker Overall?

**Figure 4:**
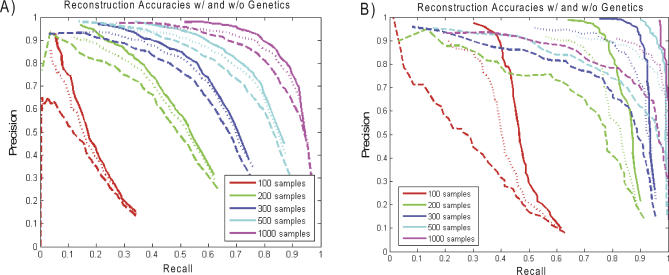
Reconstruction accuracy with the genetic (dotted and solid lines) and without the genetic (dashed lines) information, using varying numbers of samples, and based on an overall genetic signal similar to that found in the BXD network, but with weaker interactions (see text for details) (A) Reconstruction accuracy for the entire network. (B) Reconstruction accuracy for the subnetwork comprising only the top layer of the network. The dotted lines reflect reconstructions that utilized cis QTL information as the only source of genetic information, whereas the solid lines reflect reconstructions that utilized all available genetic information.

If all interactions between genes were strong, it would be relatively easy to distinguish the direct interactions from all others. Most correlations in the BXD network were strong (Figure S3), in part because the BXD network itself was reconstructed from the observed data and is therefore biased because it does not contain the weaker interactions that went undetected (due to lack of power given the modest sample size). To examine reconstruction accuracy in the presence of weaker interactions, we simulated a dataset using the same heritability (0.5) as in the BXD data, but with weaker correlations between nodes. The correlation coefficients for the gene–gene interactions were assumed to follow a normal distribution with mean 0.33 and standard deviation 0.11. Ten percent of the interactions were assumed to be nonlinear (i.e., they included a quadratic term); the correlations for the nonlinear interactions were weaker, on average, than those for linear interactions (although correlation is not an entirely appropriate measure for the nonlinear interactions). In the network as a whole, the improvement achieved by incorporating genetic information is small (Figure 4A). However, if we look only at the top layer of the network, the improvement is again much larger (Figure 4B). This is consistent with the results obtained above for the synthetic network. We have found that information on cis-acting eQTLs (excluding edges into certain nodes) and information on trans-acting eQTLs (increasing the likelihood of some edges over others) both improve the quality of reconstruction (Figure 4).

Figure S4 shows that, as intuition suggests, stronger interactions between two genes are more likely to be recovered in the reconstructed network than weaker interactions. It also shows that genetic information improves reconstruction of both weak and strong interactions.

### Question III: Is the Rate of Detection of Weak Interactions Reduced When the Overall Heritability Signature is Low?

**Figure 5:**
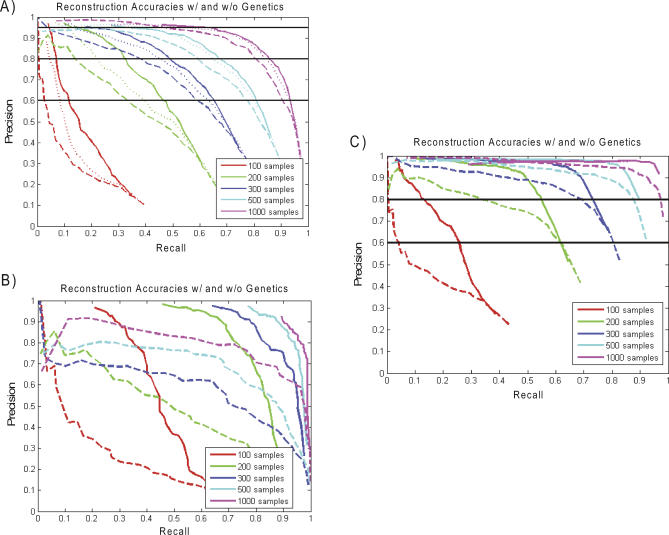
Reconstruction accuracy with the genetic (dotted and solid lines, as described in the Figure 4 legend) and without the genetic (dashed lines) information, using varying numbers of samples, and based on reduced heritability and a weak overall correlation structure compared with what we observed in the BXD network (see text for details) (A) Accuracies of networks reconstructed with and without genetic information. (B) Accuracies of subnetworks consisting only of those nodes in the top layer of the network. (C) Accuracies of networks in which a true edge was counted as correct if the corresponding nodes were connected either directly or by a path involving two edges in the reconstructed network. It is clear the genetic data significantly enhance reconstruction accuracy.

Above, we estimated the reconstruction accuracy involving weak interactions and strong heritability (50%), based on the overall heritability observed for genes in the BXD network. However, these estimates of heritability suffer from the same sort of upward bias as the correlation estimates, which are biased towards stronger correlation results. In the human population, it is rare to find a locus that explains 50% of overall trait variability. Therefore, to assess the effect of heritability on reconstruction accuracy, we simulated data in which the overall genetic heritability was set to 25%, a heritability threshold supported for genes giving rise to cis eQTL in previous studies [9], and with the weaker distribution of correlations used above (mean 0.33, standard deviation 0.11). Figure 5A shows the improvement in these reconstructions when using genetic information; Figure 5B shows that the effect of genetics is, similar to higher heritability, much stronger for the top layer of the network than for the network as a whole.

One important application of gene networks is to predict genes that will respond to changes in activity of one or more genes. We define the set of genes responding to a perturbation event in a given gene as the “signature” of the perturbed gene. For this type of application, we may be less interested in the strict accuracy of each edge and instead focus on whether genes that actually respond to a perturbation event are “near” the perturbed gene in the network. In this context, we consider an edge in the reconstructed network as correct if there is a path in the true network of length ≤2 between the perturbed node and the putative responding gene. That is, an edge is correct if the perturbed gene and responding gene are directly connected in the true network, or if they are connected with respect to a third gene. Figure 5C highlights that the genetic information improves the accuracy of network reconstruction dramatically when the data upon which the reconstruction is based obtains from a modest number of samples (100 or 200), and that, as expected, the effect of genetic information decreases for larger numbers of samples. For example, for 200 samples, nearly perfect precision was achieved (95%) at 50% recall when the genetic information was used to reconstruct the network, compared with 75% precision achieved when the genetic information was not used. The effect of genetic information using this measure of accuracy is stronger than the effect when using the stricter criterion of Figure 5A. Because networks are frequently used to predict which genes will be upregulated or downregulated in response to a single gene perturbation event, this measure of accuracy has practical relevance [5,13].

Figure S5 shows recall for different numbers of samples, with and without using genetic information, at three different levels of precision: 60%, 80%, and 95%. At the two lower levels of precision (Figure S5A and S5B), the effect of genetic information is consistent, but small. For example, adding genetic information allows us to save fewer than 100 mice for a given level of recall. However, if we restrict to high-precision edges (Figure S5C), the effect is more dramatic, where now an experiment incorporating genetic data has the potential to reduce the number of mice required to achieve a given level of recall by hundreds compared with what is required when genetic data are not used. For example, a recall of 25% is achieved using data from about 300 mice with genetic information rather than the 600–700 mice needed to achieve this same recall without genetic information. While the simulations carried out here do not perfectly represent the results that could be achieved in actual experiments, these results do suggest that incorporating genetic data improves the accuracy of reconstructed networks. Also, these results may provide some guidance on the sample sizes that may be required to achieve a certain level of accuracy in the reconstructed network with or without genetic data. At a given accuracy, incorporating genetic data may reduce the number of mice required for an experiment, which is a good in and of itself, and also saves time and money.

### Robustness of the Reconstruction Procedure

The reconstruction algorithm employed in this study uses an MCMC method, and as a result, different runs will generate different results. To test the robustness of the network reconstruction process, we compared the network reconstruction accuracy for five independent reconstructions from a single dataset comprising 100 samples (using fewer samples provides the most stringent test of robustness). To be included in a reconstructed network, an edge had to be present in at least 80% of the networks found in the individual MCMC runs. More than 85% of the edges found in any single reconstruction are found in all five reconstructions, indicating that the reconstruction procedure is quite robust. The recall and precision curves are therefore extremely similar, nearly overlapping the corresponding curves shown in Figure S6, both with and without the use of genetic data. This analysis addresses only variability arising from the stochastic nature of the reconstruction process. Additional simulations will be needed to test how stable reconstructions are to random variation in the gene expression data, which would be expected in repeated experiments measuring such data.

## Discussion

In this study, we simulated a segregating F2 population based on a biologically motivated Bayesian network (itself reconstructed from biological data derived from an actual F2 intercross population). When the simulated genotype information is not considered, the segregating population arising from an F2 cross can be treated as a randomly genetically perturbed population. We reconstructed networks in the presence and absence of the genetic data, over a range of population sizes. The results demonstrate that combining genetic and gene expression data increases the accuracy of network reconstruction. The size of the effect depends on the structure of the network: genetic information is most helpful in the “top layer” of the network. The effect of the genetic data is greater in the synthetic network compared with the biologically motivated network. This result is consistent with the results of earlier studies based on smaller networks [1]. Roughly (and intuitively), the more nodes that exist with strong genetic information, the more helpful the genetic information will be. In our simulations, we find that when high precision is required, the effect on reconstruction of adding genetic data is comparable to the effect of large increases in the amount of gene expression data available (savings of one-third to one-quarter of the subjects required, when experiments involve up to 1,000 samples). Because fewer samples are needed, and because obtaining genetic data from available samples is substantially less expensive than obtaining new samples, the use of genetic data could be a cost-effective way to improve network reconstruction.

### Connections to Other Work

There are many ways to organize data and knowledge into networks and pathways to reveal different levels of biological detail. The simplest level of biological interpretation comes from analyzing coherent gene sets, which we loosely define as sets of genes involved in common biological functions, responding to perturbations of interest, or correlating with clinical end points of interest. Gene sets are useful for high-level abstractions of biological systems, but do not provide details of the interactions among genes and between genes and external stimuli. For example, a gene set corresponding to a single gene perturbation event does not indicate which genes are primary responders to the perturbation and which are secondary responders, reacting to changes in the primary genes. At the other extreme are detailed mechanistic models, such as those based on dynamical systems [14–16]. Such models are currently only able to be constructed for relatively small, focused problems, given the large amount of data required to fit such models.

Network models fall between these two extremes. Association networks such as protein–protein interaction networks [17,18] or coexpression networks [12], examine pairwise interactions among elements of a given system. From these association networks, one can identify genes within the same highly interconnected subnetworks (gene modules) and determine how gene modules relate to specific biological functions. However, association networks do not indicate the direction of influence among genes. Probabilistic causal networks, such as Boolean, Bayesian, and probabilistic Gaussian networks, include edges with direction, and therefore can represent causal relationships among genes when causality is known. The inclusion of this information can lead to improved predictions of response to various perturbation events [19,20]. Recently, significant research interest has shifted to the use of Bayesian networks to study causal interaction networks of biological systems based on gene expression data from time series and gene knockout experiments, protein–protein interaction data derived from predicted genomics features, and other direct experimental interaction data [3,21].

Several studies have used simulations to examine network reconstruction algorithms. For example, Smith et al. [22] studied small networks (100 genes or fewer), and Yu et al. [23] studied small dynamic networks. More recently, Van den Bulcke et al. [24] proposed the SynTren scheme to combine different constraints to simulate small networks based on dynamic models. Our work is different from this previous work in many respects, but most importantly we (1) applied a unique constraint (genetics) on the data simulation, and (2) studied larger networks, including one with structure and parameters derived from biological data, allowing for more biologically realistic networks. In future work, it might be interesting to combine our data simulation scheme with SynTren [24] to generate more realistic data.

Recently, a number of studies attempting to integrate gene expression and genetic data have been published [1,5–9,19,25–27]. These studies have highlighted a number of advantages in integrating these data types to elucidate complex traits such as common human diseases, resulting in the identification of novel disease susceptibility genes. By leveraging the fact that changes in DNA segregating in experiment cross or human populations can lead to changes in transcript abundances, which in turn can lead to changes in clinical or physiological phenotypes, these approaches provide a causal anchor that previously could only be achieved by considering time series or artificial gene perturbation experiments.

### Limitations of This Study and Opportunities for Additional Research

Simulation studies are, by their nature, limited to telling us about the properties of models. To the extent a model describes a biological system of interest, the simulations can provide meaningful information. The current work makes a number of simplifying assumptions in the interest of tractability. First, many relevant biological phenomena—including, but not limited to, mRNA splice variants, protein concentrations, and protein modifications such as phosphorylation, metabolite concentrations, and noncoding RNA levels—are not represented in these models. Obviously including such data would provide for a more realistic and more complex model. In fact, our model does not even include all of the protein-coding genes known in the mouse genome, but instead restricts attention to the approximately 2,000 genes found to be most differentially regulated in the biological samples upon which our simulations are based [5]. We accept the limitations in this current study in hopes of taking the necessary first steps to systematically assess what the genetic information can bring to the gene network reconstruction problem, even if the gene network contains only a fraction of the known functional units in the genome. Evaluating the difference more realistic models make will require generation of more comprehensive and larger-scale datasets, in addition to creating and analyzing models that take more of the biological information into account.

Second, the distribution of eQTL for nodes in the network allowed only head nodes to be assigned cis eQTL, and the head nodes were assumed to have expression perturbations corresponding to eQTL as the ultimate causal events. In practice, the distribution of eQTL is more complex, with genes driven by cis eQTL also driven by potentially complicated networks of trans eQTL that could involve epistatic interactions. Getting a handle on the overall genetic variance component for each expression trait will be an important next step that has only recently begun to be addressed [6,28,29]. Also, there may be changes in DNA that lead to protein state changes or other such changes that don't necessarily result in expression changes of the corresponding gene, so that in such cases the ultimate causal event would not be DNA changes in a gene leading to changes in expression of that gene, as we have modeled here.

Third, we employed Bayesian networks in this study. Bayesian networks do not permit loops, making it difficult to represent some types of feedback. Bayesian networks also do not represent time-series data well [30]. Both of these problems might be addressed by using dynamic Bayesian networks [31], which explicitly allow for a temporal representation of how nodes in the network interact with one another. We plan to address the use of dynamic Bayesian networks in future studies. In addition, we examined reconstruction of only a single biologically motivated network structure, and a single synthetic network with a simpler structure. Results demonstrate that using genetic data helps reconstruct both the biologically motivated and the synthetic network to different degrees. How network structure affects reconstruction accuracy will require further investigation.

The results reported herein provide some of the first estimates regarding the benefits in experimental design of combining genetic and gene expression data to reconstruct predictive gene networks. However, despite incorporating information derived from actual biological data, the models simulated in this study were necessarily much less complicated than the actual biological systems they approximate. One of the consequences of reducing model complexity to make these simulations possible is underestimating the amount of biological data that may be needed to construct predictive networks. Therefore, our simulations should not be used at this point to determine how many biological samples are actually needed to reconstruct a network with a given level of accuracy. Rather, the relationship between network accuracy and sample size reported here provides more of a lower bound on the number of samples that may be required and highlights that integrating genetic and gene expression data can lead to more reliable gene networks. The integration of additional types of data (for example, ncRNA levels, protein levels, protein–protein interaction, protein–DNA binding, protein state, and so on) in addition to genetic and expression data will likely allow for further improvements in network reconstruction with the same number of biological samples.

### Conclusion

Ever larger sets of data are being generated and integrated at a furious pace in all areas of biology. Inevitably, modeling and computation will play a fundamental role in understanding these data. Network approaches, which consider how all components of a system are connected, have the potential to capture critical features of the data. It is important to remember, however, that any network is just the result of fitting a particular mathematical model to a given set of data. Therefore, just as with any mathematical model, we must examine the quality of the fit of the data to the model to determine whether or not the model adequately represents the system under study. One of the best ways to evaluate the effectiveness of different network reconstruction methods using different types of data is to apply them in situations where the correct answer is known. While we cannot generally at this point carry out this type of evaluation using biological data (what is known is simply too incomplete at this time), we can do it using data simulated from a known model, as demonstrated here. The ability to compare models in a systematic fashion will let us determine which data types and reconstruction methods produce the most reliable and predictive network representations of the data. The work presented here provides some of the initial steps needed to assess in a more systematic fashion how integrating different types of data can enhance network reconstruction and what sample sizes may be required to realize more reliable, more predictive gene networks.

## Materials and Methods

The information flow diagram for data simulation and network reconstruction is shown in Figure 1. The main steps in the process are outlined below.

### Simulating a segregating population.

An F2 intercross population was simulated using the QTL Cartographer suite of software tools [32]. Given a genetic map, the genetic models (which include QTL location, and the additive, dominance, and epistatic effects) for the gene expression traits were simulated using the Rqtl tool [32]. The genetic map used in this study was constructed from a previously described F2 intercross population constructed from the C57BL/6J and C3H/HeJ strains of mice [33]. This genetic map consisted of 1,357 markers for 19 autosomal chromosomes. Rqtl was configured such that, on average, one QTL was simulated for each gene expression trait (via the “*−q 1*” option in the Rqtl tool). Dominance was assumed to be random with respect to direction and magnitude for each locus. Given the simulated QTL for each of the expression traits, the F2 population (including genotypes for each marker and trait values for each head node) was then simulated using the QTL Cartographer Rcross procedure. The heritability for each expression trait was set to 0.5 in addressing questions I and II, and to 0.25 for addressing question III, as defined above.

### Network structure.

In a network with *n* nodes, there are approximate *n^n^* possible structures. Even if we impose restrictions on network structure based on observed properties of biological networks, such as limiting the number of parent nodes [15], restricting the frequency of certain structural motifs [34], or requiring that the network be scale-free [35], the number of possible structures will be intractably large. Sampling different network structures systematically and studying how structure affects network reconstruction are outside the scope of this paper. Here we examine two types of networks, a biologically motivated network reconstructed from the BXD data (see Methods for details), and a synthetic network consisting of a set of simple isolated structures.

### Simulating expression data.

There are two types of nodes in a Bayesian network: nodes without parents (head nodes) and nodes with parents (non-head nodes). As shown in Figure 1, we used different approaches to generate values for the two types of nodes. Traits assigned as head nodes were simulated based on QTL location and heritability, as defined above [36]. After the values of the head nodes were determined, the values of non-head nodes were constrained by the strength of the gene–gene interactions and the values at the head nodes. Gene–gene interactions in Bayesian networks are described by a set of conditional probability functions. The conditional probabilities can be specified for continuous values (e.g., between means of the Gaussian distributions for each gene) or for discrete values representing the possible states for each gene.

We simulated data by considering the nodes as normally distributed random variables. Gene–gene interactions were allowed to be linear as well as nonlinear, given sigmoidal and other such nonlinear relationships have been observed between gene expression traits in a number of settings [37]. For two-channel gene expression microarray experiments, relative expression levels are often represented as log ratios, where the ratio is defined as the experimental channel divided by the reference channel. The log function in this case can transform some nonlinear relationships into linear ones [38]. Therefore, to simulate gene expression traits for the purpose of modeling transcription regulatory networks, we assumed that 90% of the interactions were linear, as defined above. For a given gene expression trait *y,* interactions with other expression traits were simulated as:

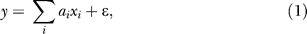
for linear interactions, and as:

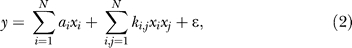
for nonlinear interactions (which were limited to second-order terms), where the *x_i_* are the gene expression traits interacting with *y, a_i_* the additive regression coefficients, *k_ij_* the nonlinear regression coefficients, and *ɛ* the noise term, assumed to be normally distributed with mean 0 and variance *σ* ^2^. The coefficients in this case are constrained by the strength of correlation (either fixed or sampled from a distribution, as described in the Results section), defined as *corrcoef*(*y*,*x_i_*) = *ρ_i_*. The sign of the coefficient *a_i_* was randomly assigned, but the overall numbers of positively and negatively correlated pairs were constrained to be equal. Because we take the random vector (*Y*, *X*_1_, …, *X_N_*) to be jointly normally distributed, we can write the covariance for this random vector as 
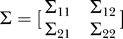
 , where Σ_11_ is of size 1 × 1, Σ_12_ is of size 1 × *N*, Σ_21_ is of size *N* × 1, and Σ_22_ is of size *N* × *N*. The regression coefficients, *a_i_*, in Equations 1 and 2 are then given by 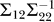 , and the error term, *ɛ,* is normally distributed with mean 0 and variance 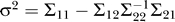 . The *k_i_* in Equation 2 were randomly sampled from a normal distribution with mean 0.50 and variance 0.15.

To address questions I and II defined in the Results section, the average heritability was estimated from the BXD data, while the average correlation between nodes was taken to be significantly weaker than what was observed in the BXD data. To address question III, the average heritability was decreased compared with that observed in the BXD data to better generalize the type of results that may be realized in applying network reconstruction methods in other settings (e.g., human populations).

### Bayesian network reconstruction.

Bayesian networks are directed acyclic graphs in which the edges of the graph are defined by conditional probabilities that characterize the distribution of states of each node given the state of its parents [2]. The network topology defines a partitioned joint probability distribution over all nodes in a network, such that the probability distribution of states of a node depends only on the states of its parent nodes: formally, p(node j | all non-descendants of node j) = p(node j | parents(node j)). These conditional probabilities reflect not only relationships between genes, but also the stochastic nature of these relationships, as well as noise in the data used to reconstruct the network. Because the edges in Bayesian networks are directed, they not only represent interactions among genes, but also, when such information is available, they can represent causal associations between genes. Therefore, these probabilistic networks allow us to predict the system's response to perturbations based on the identified relationships among genes.

Bayes formula allows us to determine the probability of a network model M given observed data D as a function of our prior belief that the model is correct and the probability of the observed data given the model: P(M | D) ∼ P(D|M) * P(M). The number of possible network structures grows super-exponentially with the number of nodes, so an exhaustive search of all possible structures to find the one best supported by the data is not feasible, even for a relatively small number of nodes. We employed Monte Carlo Markov Chain (MCMC) [39] simulation to identify potentially thousands of different plausible networks, which are then combined to obtain a consensus network (see below). Each reconstruction begins with a random network. Small random changes are then made to the network by flipping, adding, or deleting individual edges, ultimately accepting those changes that lead to an overall improvement in the fit of the network to the data. We use a uniform prior probability on networks and assess whether a change improves the network model using the Bayesian Information Criterion (BIC) [40], which avoids overfitting by imposing a cost on the addition of new parameters. This is equivalent to imposing a lower prior probability P(M) on models with larger numbers of parameters.

In this study, for each dataset we reconstructed 1,000 Bayesian networks starting with 1,000 different randomly generated Bayesian networks (seeds). MCMC simulation was then employed to identify the most plausible networks. For each seed, 15 × *N* ^2^ iterations of MCMC were run (on average the maximum BIC scores were reached at roughly 12 × *N* ^2^ iterations), where *N* is the number of nodes in the network. From the 1,000 reconstructed networks we determined the consensus network by retaining only those edges represented in a percentage of the individual networks. A ROC curve can be generated by varying the percentage threshold. Increasing the percentage threshold for inclusion leaves fewer, but more reliable edges in the consensus network (see Results), presenting the usual tradeoff between sensitivity and specificity. Because Bayesian networks must be acyclic, loops in the consensus network were removed by deleting the weakest links in the loops [5].

Gene expression was simulated as a continuous random variable, then discretized into one of three possible states (downregulated, upregulated, or no change relative to the reference channel) guided by k-means clustering, modified to discourage extremely unbalanced classes, with the number of clusters allowed for a given gene over all individuals in the F2 population set to three. A Bayesian network was reconstructed based on the discretized data. There are two primary reasons for reconstructing Bayesian networks using discretized data. First, only linear interactions can be modeled under the Gaussian model for continuous data, whereas with discretized data Bayesian networks can capture nonlinear interactions. Second, network reconstruction using discrete data is much faster than reconstruction using continuous data.

### Genetic data as a network prior.

In general, Bayesian network structures can only be solved to Markov equivalent structures. That is, it may not be possible to determine the direction of many edges. The reconstruction algorithm employed can take advantage of the experimental cross design (or segregating populations more generally) by incorporating genetic data [5]. If genetic information is not available or is ignored, the population is simply treated as a population with random genetic perturbations. In the present algorithm, we use the genetic data as prior evidence that two genes may be causally related. There are three sources of genetics priors. First, genes with cis-acting eQTLs [10] are allowed to be parent nodes of genes with trans-acting eQTLs, *p*(*cis* → *trans*) = 1; but genes with trans-acting eQTLs cannot be parents of genes with cis-acting eQTLs, *p*(*cis* → *trans*) = 0. Second, genes with suggestive eQTLs [41] (LOD scores greater than 2.8, corresponding to a point-wise *p*-value of 0.001) were identified. Genes from the set of genes with cis or trans eQTL were then tested individually for pleiotropic effects at each of their eQTL to determine whether any other genes in the set were driven by the same eQTLs [42]. If yes, the gene pair and the locus where they have a pleiotropic effect are used to infer a causal/reactive or independent relationships based on a formal causality test [1]. The reliabilities of the inferred relationship between gene A and gene B at locus *l_i_*, *p*(*A* → *B*| *A*,*B*,*l_i_*), *p*(*B* → *A* | *A*,*B*,*l_i_*), and *p*(*A*⊥*B* | *A*,*B*,*l_i_*), are estimated by a standard bootstrapping procedure. If an independent relationship is inferred *p*(*A*⊥*B* | *A*,*B*,*l_i_*) > 0.5, then the prior probability that gene A is a parent of gene B is scaled as

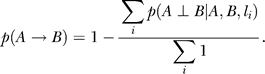
If a causal or reactive relationship is inferred (p(A → B| A,B,li) or p(B → A | A,B,li) is greater than 0.5), then the prior probability is scaled as

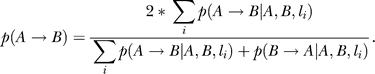



Third, if the causal/reactive relationship between genes A and B cannot be determined by the above two sources, then the complexity of the eQTL signature for each gene was taken into consideration. Genes with a simpler, albeit stronger eQTL signature (i.e., a small number of eQTL explains the genetic variance component for the gene, with a significant proportion of the overall variance explained by the genetic effects) were taken to be more likely to be causal compared with genes with more complex and possibly weak eQTL signatures (i.e., a larger number of eQTL explaining the genetic variance component for the gene, with less of the overall variance explained by the genetic effects). The structure prior that gene A is a parent of gene B is

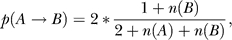
where n(A) and n(B) are the number of eQTLs with LOD scores greater than 2.8 for A and B, respectively. We have found that both information on cis-acting eQTLs (excluding edges into certain nodes) and information on trans-acting eQTLs (increasing the likelihood of some edges over others) improve the quality of reconstruction.

In actual experiments, the estimated error rate associated with determining cis-acting eQTLs [10,13] is low. A genome-wide scan of 25,000 gene expression traits, using a LOD score cutoff of 7 (point-wise *p*-value = 1.02 × 10^−7^), is expected to yield 0.03 false cis-acting eQTLs by chance (1.02 × 10^−7^ × 25,000 genes × 10 markers tested around the gene's physical location). Because we do not simulate genomic information in this study, we cannot use genomic and expression data to detect cis-acting eQTLs in the simulated data. Instead, a percentage of the head nodes are simply designated as cis-acting eQTLs, while the others are designated as trans-acting. Several studies have shown that roughly 5% of genes in a given experimental cross comprised several hundred animals and in a single tissue give rise to cis-acting genes [9,10]. In addition to genes with cis eQTL, other genes can also serve as head nodes in the Bayesian networks. In our simulation, 64% (70/110) of the nonsingleton head nodes (4.6% of the nonsingleton nodes in the network) and 7% (45/639) of the singleton nodes were designated as giving rise to cis-acting eQTLs.

### Reconstruction of correlation-based association networks.

Like Bayesian networks, correlation-based association networks are graphical models in which the nodes are genes and edges between the nodes indicate a significant correlation between the corresponding expression traits in a given tissue of interest. Unlike Bayesian networks, the edges in a correlation-based association network are undirected. To define a gene–gene correlation-based association network, two gene expression traits (nodes) were considered linked (connected by an edge) if the *p*-value for the Pearson correlation coefficient between the two genes was less than some pre-specified threshold. To reconstruct the ROC-like curves for the correlation networks depicted in Figure S7, we varied the correlation coefficient *p*-value threshold used to determine whether two nodes should be connected by an edge.

### Comparing the reconstructed network with the true network: Recall and precision.

To assess the goodness of the reconstructed network, it was compared with the true network used in the simulation process. We define the “goodness” of the reconstructed network in terms of its accuracy, which is measured by two parameters. The first parameter is defined as the precision of the network:

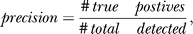
which is the proportion of detected interactions that actually exist in the true network. Precision corresponds to specificity and is equal to one minus the false positive rate (1 − *FPR*). The second parameter is defined as the recall of the network:

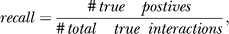
which is the proportion of total interactions in the true network that are detected in the reconstructed network. Recall corresponds to sensitivity and is equal to one minus the false negative rate (FPR) (1 − *FPR*), which is also known as the true positive rate (*TPR*). The recall and precision for a perfectly reconstructed network are equal to 1.

Throughout the text, true positive events are defined in three separate ways, depending on the context: (1) since Bayesian networks are directed graphs, the most stringent way to define a true positive event is to require that not only the link but also the direction between two nodes in the constructed network be the same as the true network; (2) if we ignore the direction of the edges in a Bayesian network, true positive events are then the edges that exist in both the constructed and true networks; and (3) whether two nodes interact directly or indirectly through a third node is not important in some applications (e.g., when a network is used to predict a responder or a biomarker set, genes responding to a perturbed node [1,5,27] can be directed or undirected), and therefore, in such cases, interactions in the reconstructed networks can be considered as true positive events if the two nodes interact directly or indirectly in the true network.

### Comparing the network reconstruction methods: ROC curves.

The central figure of merit used to evaluate and compare the different reconstructed networks to each other (with respect to the true network) is the recall versus precision curve, which can be considered as a variation of the traditional ROC curve. ROC curves are generated by plotting the true positive rate (TPR) against the false positive rate (FPR). The area under the ROC curve (AUC) is then a measure of how the constructed network compares with the true network. The larger the AUC, the better the constructed network compares with the true network, where the maximum AUC is 1, indicating that the constructed network perfectly matches the true network. As defined above, recall is equivalent to the TPR, while precision is equal to 1 − *FPR*. Therefore, the recall vs. precision curve is generated by plotting 1 − *FPR* against the TPR. The recall versus precision curve has the same characteristics as the ROC curve in that the AUC is a measure of how well the constructed network compares with the true network, with an AUC of 1 indicating a perfectly reconstructed network. Qualitatively, the recall versus precision curve is equivalent to the ROC curve in that if the AUC for one network is greater than (or less than) the AUC of a second network with respect to one of plot types, that same relationship will hold for the other plot type. We opted to use the recall versus precision plots over the ROC plots as the figure of merit because recall and precision are the more standard measures used in the network reconstruction community.

## Supporting Information

Figure S1 General Properties of the BXD Network Structure Used in the Simulation Study (A) Most genes in the network have only one parent.
(B,C) Log–log plots of number of genes with multiple children or connections. The network has some general features of a scale-free network.
(D) A global view of the whole network shows that most of genes are interconnected.
(6.2 MB EPS)

Figure S2 The Synthetic Structure Consists of 360 Isolated Three-Node Causal/Reactive Networks and 360 Isolated Three-Node Independent Models This gives 2,160 nodes and 1,440 interactions, similar to the number in the BXD network. The root nodes (squares) are assumed to have cis-ating eQTLs.
(1.1 MB EPS)

Figure S3 Properties of Simulated Data (A) Histogram of correlation coefficients of interacted genes in BXD dataset.
(B) Histogram of QTL peaks. The peaks of head nodes are evenly distributed. The peaks of QTLs for all nodes are clustered into several hot spots, similar to the BXD data [9].
(1.5 MB EPS)

Figure S4 Fraction of Interactions Recovered with 60% Precision at Different Interaction Strength The left and right columns show results for the BXD network and for the synthetic network (using 100 samples), respectively.
(Top) Number of interactions with different correlation strengths. Each point shows results for edges with correlation strength within 0.05 on either side of the indicated value.
(Bottom) Fraction of edges of each strength detected, with genetic information (solid) and without genetic information (dashed).
(1.4 MB EPS)

Figure S5 Recall versus Number of Samples at Different Levels of Precision (A,B,C) 60%, 80%, and 95%, respectively. At the two lower levels of precision, adding genetic information gives relatively modest gains in recall, while at 95% precision, the increase in recall from genetic data is substantial. The curves based on 95% precision are not smooth in places, especially for the networks reconstructed without the genetic data. This lack of smoothness is due to the fact that a large section of each curve is parallel to the 95% precision threshold line (see Figure 5A). As a result, the exact points at which the 95% precision threshold line and the ROC-like curves intersect are sensitive to small variations in the ROC-like curves.
(2.4 MB EPS)

Figure S6 Robustness of Reconstruction Accuracies of the BXD Bayesian Network Using 100 Gene Expression Measurements with and without Genetics The results of five independent runs indicate the reconstruction process is stable even though the procedure is stochastic in nature.
(A) Reconstruction accuracies of five independent runs.
(B) Reproducibility of individual predicted interactions at different thresholds.
(2.0 MB EPS)

Figure S7 ROC Curves Showing Accuracy of Reconstructions of the Simulated BXD Network Ignoring Edge Direction (Dashed Lines) and Considering an Edge as Correct if the Nodes Are Connected by a Path of Length Less Than or Equal to 2 (Solid Lines) Three methods are shown: Bayesian networks without genetic information (red lines), Bayesian networks with genetic information (green), and association networks based on correlation (red). The curve for the correlation-based association network was generated by varying the correlation coefficient *p*-value threshold used to establish whether or not two nodes are connected by an edge (see the Methods section for details).
(777 KB EPS)

## Abbreviations

eQTL: expression quantitative trait loci
